# Prediction of Metabolic Parameters of Diabetic Patients Depending on Body Weight Variation Using Machine Learning Techniques

**DOI:** 10.3390/biomedicines13051116

**Published:** 2025-05-04

**Authors:** Oana Vîrgolici, Daniela Lixandru, Andrada Mihai, Diana Simona Ștefan, Cristian Guja, Horia Vîrgolici, Bogdana Virgolici

**Affiliations:** 1 Academy of Economic Studies, 010374 Bucharest, Romania; oanavirgolici2022@gmail.com; 2 Faculty of Midwifery and Nursing, ”Carol Davila” University of Medicine and Pharmacy, 050474 Bucharest, Romania; daniela.lixandru@umfcd.ro; 3 Faculty of Medicine, ”Carol Davila” University of Medicine and Pharmacy, 050474 Bucharest, Romania; andrada.mihai@gmail.com (A.M.); cristian.guja@umfcd.ro (C.G.); bogdana.virgolici@umfcd.ro (B.V.); 4 National Institute of Diabetes, Nutrition and Metabolic Disease “N. Paulescu”, 020475 Bucharest, Romania; simona_ds2002@yahoo.com

**Keywords:** machine learning, body mass index, diabetes mellitus, diet and nutrition, metabolic health, weight loss

## Abstract

**Background/Objectives**: Obesity is a major risk factor for diabetes mellitus, a metabolic disease characterized by elevated fasting blood glucose and glycosylated hemoglobin levels. Predicting the percentage and absolute variations in key medical parameters based on weight changes can help patients stay motivated to lose weight and assist doctors in making informed lifestyle and treatment recommendations. This study aims to assess the extent to which weight variation influences the absolute and percentage changes in various clinical parameters. **Methods**: The dataset includes medical records from patients in Bucharest hospitals, collected between 2012 and 2016. Several machine learning models, namely linear regression, polynomial regression, Gradient Boosting, and Extreme Gradient Boosting, were employed to predict changes in medical parameters as a function of body weight variation. Model performance was evaluated using Mean Squared Error, Mean Absolute Error, and R^2^ score. **Results**: Almost all models demonstrated promising predictive performance. Quantitative predictions were made for each parameter, highlighting the relationship between weight loss and improvements in clinical indicators. **Conclusions**: Weight loss led to significant improvements in dysglycemia, dyslipidemia, inflammation, uric acid levels, liver enzymes, thyroid hormones, and blood pressure, with reductions ranging from 5% to 30%, depending on the parameter.

## 1. Introduction

Obesity is a major risk factor for the onset of diabetes mellitus. Diagnosis and monitoring of this metabolic disorder rely on glycosylated hemoglobin (HbA1c) levels and fasting blood glucose measurements. According to the American Diabetes Association guidelines, an HbA1c level of 6.5% or higher is a diagnostic criterion for diabetes mellitus [1]. Sustainable weight loss in obese individuals can enhance glucose metabolism and contribute to the remission of type 2 diabetes mellitus [2]. A 2012 study demonstrated that a medically supervised weight loss program combining diet and exercise effectively induces weight loss, lowers fasting blood glucose levels in both diabetics and non-diabetics, and reduces HbA1c levels in individuals with diabetes. The observed 0.8% reduction in HbA1c was clinically significant in preventing diabetes-related complications [3]. Additionally, the United Kingdom Prospective Diabetes Study found that a 1.0% reduction in HbA1c was associated with significant improvements in both morbidity and mortality [4].

Weight loss interventions are crucial for managing obesity, prediabetes, and type 2 diabetes. Studies have shown that such interventions can effectively reduce fasting blood glucose levels [5]. As β-cell function declines in obesity and diabetes, modifications in diet and medical treatment become essential. Early glycemic control helps mitigate β-cell exhaustion and prevent complications. Research further supports the efficacy of weight loss medications and metabolic surgery in improving metabolic outcomes [6]. Additionally, significant intentional weight loss has been shown to reduce the risk of cardiovascular disease in individuals with overweight or obesity [7].

Machine learning (ML) methods have proven effective in predicting obesity [8]. Additionally, ML plays a crucial role in metabolomics by assisting in data preprocessing and the development of predictive models for disease forecasting, biomarker identification, phenotyping, metabolic profiling, and dietary assessment [9].

The objective of this study is to evaluate the impact of body weight changes on both absolute and percentage variations in key health parameters used to assess patients’ health status. Additionally, this study aims to predict how these parameters are likely to respond to weight fluctuations. These predictions can provide valuable insights to both healthcare providers and patients in managing medical conditions, particularly type 2 diabetes mellitus.

## 2. Materials and Methods

The dataset used in this study comprises medical information from over 100 patients treated at the National Institute of Diabetes, Nutrition and Metabolic Diseases ‘N. Paulescu’ in Bucharest. These patients, all diagnosed with type 2 diabetes within the previous six years and undergoing chronic medication, were monitored over time. Patients were classified based on their body mass index (BMI). Those with a BMI of 25–30 kg/m^2^ were categorized as overweight (44%), while those with a BMI ≥ 30 kg/m^2^ were categorized as obese (56%).

The American Diabetes Association (ADA) recommends an energy intake of 25–30 kcal/kg/day to achieve a 5–10% weight loss in people with overweight or obesity and type 2 diabetes. Nutrition, physical activity, and behavioral therapy are advised to maintain at least 5% weight loss. Intensive interventions with ≥16 sessions in 6 months, focusing on nutrition, physical activity, and creating a 500–750 kcal/day deficit, are strongly recommended for effective weight loss. Caloric needs should be individualized based on factors such as weight, age, sex, and physical activity level [10].

Meta-analyses show no clear superiority of any macronutrient profile in hypocaloric diets for type 2 diabetes. Very low energy diets and formula meal replacements are most effective, especially for remission, but evidence is mostly limited to under one year [11].

Medical data were collected at two time points: initially, starting in early 2012, and again through 2016. Throughout the study period, each patient’s medication regimen remained unchanged. However, all participants were advised to adopt a weight loss strategy based on a caloric deficit of 30 kcal/kg per day. Communication with patients during the monitoring period was conducted by phone every three months to encourage them to adhere to the diet.

Each patient served as their own control, with weight comparisons made between the beginning and end of the study. Exclusion criteria included a history of congestive heart failure, myocardial infarction, or stroke within the previous six months, severe liver or kidney disease, and malignancy.

The dataset includes demographic information (name, sex, and age), anthropometric measurements (weight and height), and blood pressure readings, which are categorized as follows:

(a) Dysglycemia parameters: HbA1c, fasting blood glucose, and HOMA-IR (Homeostatic Model Assessment for Insulin Resistance).
(b) Dyslipidemia parameters: total cholesterol, HDL cholesterol, and triglycerides.
(c) Inflammation parameters: C-reactive protein (CRP), IL-6 (Interleukin-6), TNF-α (Tumor Necrosis Factor α), leptin, and adiponectin.
(d) Liver function parameters: AST (Aspartate Aminotransferase) and ALT (Alanine Aminotransferase).
(e) Kidney function parameters: uric acid and creatinine.

For each analyzed parameter, only the records of patients with both baseline and follow-up measurements were retained.

After an overnight fast, blood samples were collected in plain vacuum tubes for routine laboratory tests and ELISA measurements, or in EDTA-containing tubes for HbA1c assays. This study was approved by the Ethics Committee of the National Institute of Diabetes, Nutrition, and Metabolic Diseases ‘N. Paulescu’ (Approval No. 5/22.11.2011), Bucharest, Romania. Informed consent was obtained from all participants.

HbA1c was measured using standard HPLC techniques from fasting venous blood samples. Insulin levels were assessed using the EIA-2935 kit (intra-assay CV: 2.2%) and leptin levels using the EIA-2395 kit (intra-assay CV: 6.43%), both from DRG Instruments GmbH, Marburg, Germany. TNF-α concentrations were measured using Cayman ELISA kits (No. 589201, Cambridge, UK), with an intra-assay CV of 5.9% and an inter-assay CV of 9.8%. Plasma samples were analyzed in triplicate using a Multiskan EX Thermo device, Thermo Fisher Scientific, Waltham, MA, USA for ELISA assessments.

To optimize feature selection and analyze the impact of weight loss on health parameters, we calculated the percentage changes in weight and percentage or absolute changes for each studied parameter, creating a new dataset consisting of these values. This dataset was then used to apply regression algorithms, allowing us to assess the relationship between changes in weight and the corresponding changes in relevant parameters. In the new datasets, only the records from the original dataset that contained both initial and final values were used. In other words, incomplete records were removed.

Data preparation, after retaining complete records, involved data cleaning and the application of the following steps, with initial and final scatter plots generated for exploratory purposes:

(a) Initial scatter plot: a preliminary visualization of the relationship between the two variables, providing an overview of the data distribution.
(b) Outlier detection based on residuals: Identifies observations where the residuals (differences between observed and predicted values) exceed ±3 standard deviations (3σ), indicating potential outliers. An outlier is also defined as any data point where the signs of the variation in an analyzed parameter and the variation in body weight are discordant. All parameters align with this rule, except for HDL cholesterol, where, from a medical standpoint, the two variations are expected to have opposite signs.
(c) Influence analysis via Cook’s distance: evaluates the influence of each observation on the fitted regression model, highlighting data points that disproportionately affect the model’s accuracy.
(d) Automatic removal of problematic points: automatically filters out data points identified as outliers based on large residuals, a high Cook’s distance, or discordant signs between variables.
(e) Final scatter plot (cleaned data): displays the dataset after removing the problematic observations, showcasing the cleaned data distribution.
(f) Returning two data frames: the dataset with outliers removed, now prepared for regression analysis, and the problematic dataset, which consists of a separate collection of the detected outliers, which can be analyzed further if needed.

For example, for the data-cleaning process, which involved the relationship between the percentage variation in blood glucose and the percentage variation in body weight, out of the initial dataset containing 136 observations, 12 outliers were identified. As a result, the final dataset prepared for the application of machine learning algorithms contained 123 observations, representing a removal of 8.88% of the initial data points. This percentage of removed observations is considered acceptable for ensuring the scientific integrity of the analysis, as it aligns with common practices in data preprocessing.

Given that the variation in each analyzed parameter in relation to the percentage change in body weight may follow either a linear or a more complex pattern, machine learning techniques were employed within custom Python 3.7.7 64-bit applications to model these relationships accurately. The methods applied included linear regression, polynomial regression, Gradient Boosting, and Extreme Gradient Boosting.

The hyperparameter optimization was conducted using RandomizedSearchCV and GridSearchCV for regression tasks with both Gradient Boosting and XGBoost models. Further details are provided in the subsequent section.

Data visualizations were generated using the matplotlib.pyplot module. Model performance was evaluated using the following metrics: Mean Squared Error (MSE), Mean Absolute Error (MAE), and the R^2^ score.

## 3. Results

To illustrate the methodology without overloading the paper with results for all dataset parameters, we present in detail the absolute variation in HDL cholesterol as a function of the percentage variation in body weight as a representative example. However, the discussion and final conclusions are based on the analysis of all parameters included in the study.

HDL cholesterol is an important parameter for the diagnosis of metabolic syndrome and cardiovascular disease risk. The HDL cholesterol dataset had 107 records.

### 3.1. Liniar and Polynomial Regression

**Figure 1:**
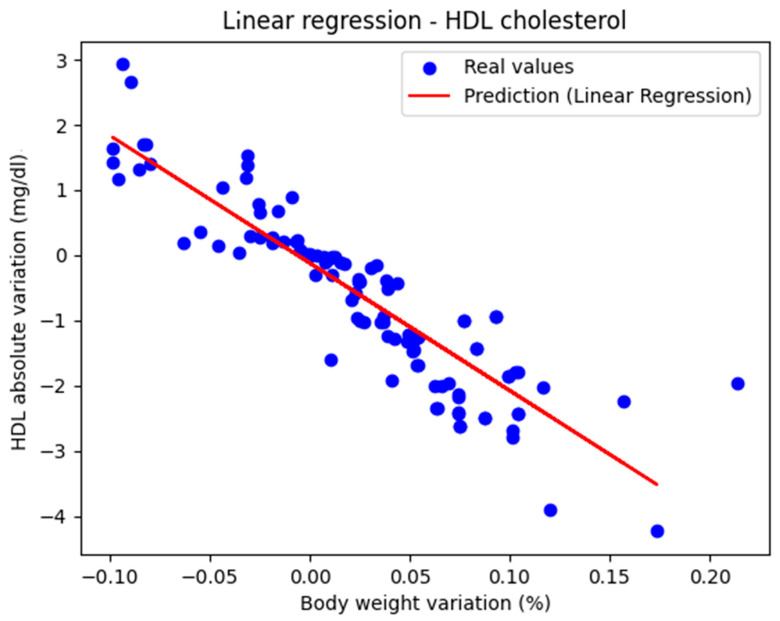
Actual values and predicted values using linear regression showing the relationship between the absolute variation in HDL cholesterol and the percentage variation in body weight.

Figure 1 shows the representation of the actual values along with the linear regression line. The performance metrics were as follows: MAE = 0.447, MSE = 0.434, and R^2^ score = 0.770, indicating that the model can be considered very good.

The regression line has a negative slope, which is medically correct, because HDL cholesterol is the parameter whose value is considered ‘better’ as it increases. The second-degree polynomial had the coefficients [0. −21.872 35.480]

The model’s performance was characterized by the following metric values: MAE = 0.441, MSE = 0.263, and R^2^ score = 0.801. Thus, it slightly outperformed the linear regression model. The conclusion is that both linear and polynomial regression models approximate the real values of HDL cholesterol from good to very good.

Table 1 summarizes the performance of the linear regression and polynomial regression models in estimating the absolute variation in HDL cholesterol based on the percentage variation in weight. While polynomial regression yielded a better R^2^ score, both models can be considered good to very good.

### 3.2. Gradient Boosting

With only 107 records in our dataset, the risk of overfitting and poor generalization increased when using Gradient Boosting.

**Figure 2:**
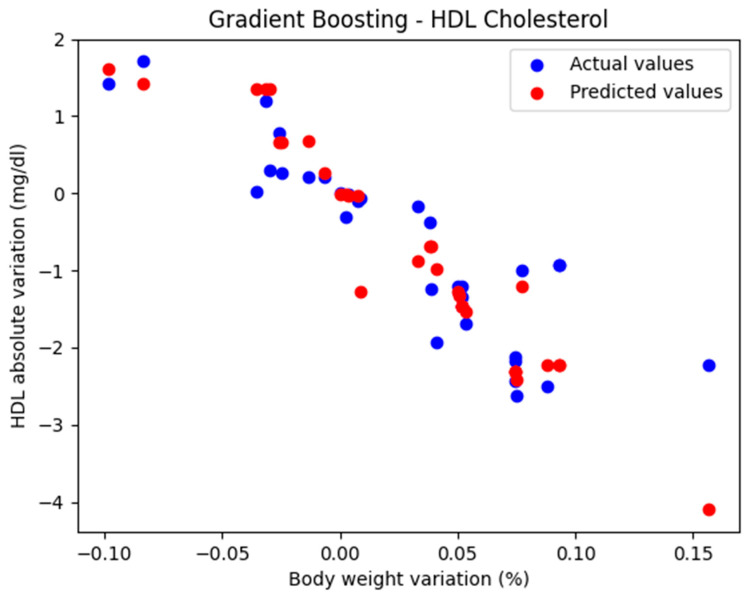
Comparison between actual and predicted values using Gradient Boosting.

In the initial approach, the GradientBoostingRegressor function was used with the default parameters: n_estimators = 100, learning_rate = 0.1, and max_depth = 3. The comparison between the actual and predicted values using the Gradient Boosting model, for the analysis of the absolute variation in HDL cholesterol relative to the percentage variation in body weight, is illustrated in Figure 2. The model evaluation yielded the following results: MAE = 0.437, MSE = 0.424, and R^2^ = 0.694. These findings support the classification of the model as good.

#### 3.2.1. Optimized Gradient Boosting Model Through GridSearchCV

GridSearchCV is a powerful tool for hyperparameter optimization and for creating a more robust model. Given the small size of the dataset, hyperparameter tuning was performed using GridSearchCV with modest values for the hyperparameters ‘n_estimators’: [50, 100], ‘learning_rate’: [0.01, 0.05], ‘max_depth’: [3, 4], ‘min_samples_split’: [2, 5], ‘min_samples_leaf’: [2, 5], and ‘subsample’: [0.7, 0.8].

The best hyperparameters obtained for the Gradient Boosting model through GridSearchCV were {‘learning_rate’: 0.05, ‘max_depth’: 4, ‘max_features’: None, ‘min_samples_leaf’: 2, ‘min_samples_split’: 10, ‘n_estimators’: 50, ‘subsample’: 0.7}.

**Figure 3:**
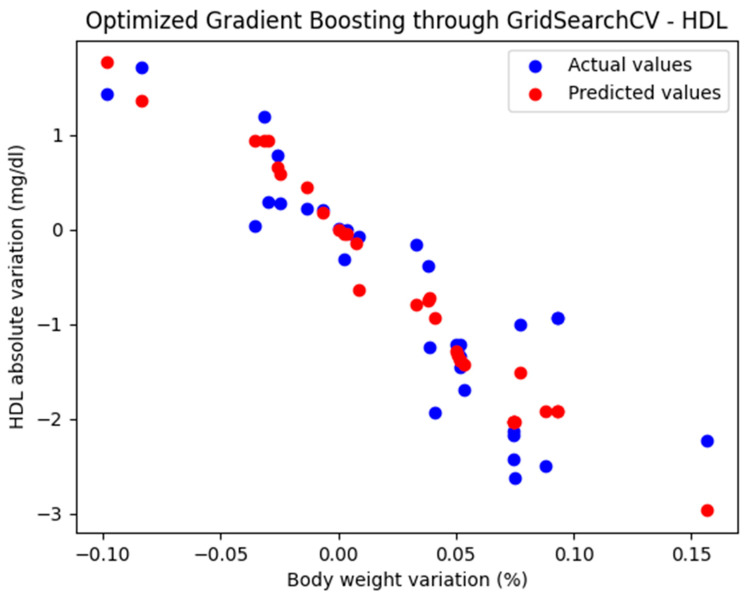
Comparison between the actual and predicted values obtained with the optimized Gradient Boosting model through GridSearchCV.

For these hyperparameters, the graphical representation of the actual and predicted values obtained using the optimized Gradient Boosting model through GridSearchCV is shown in Figure 3.

The optimized Gradient Boosting model, through GridSearchCV, yielded the following performance metrics: MAE = 0.374, MSE = 0.233, and R^2^ = 0.825. With an R^2^ score of 0.885, the model demonstrated a very good fit and strong predictive performance.

#### 3.2.2. Optimized Gradient Boosting Model Through RandomizedSearchCV

RandomizedSearchCV randomly tests a predefined number of combinations. It is faster and, in some cases, sufficiently effective.

Due to the small dataset, a higher number of folds were used (cv = 10). RepeatedKFold was used to stabilize the result, and a relatively small number of combinations were set (n_iter = 10).

The best hyperparameters obtained for the Gradient Boosting model through RandomizeSearchCV were {‘subsample’: 0.7, ‘n_estimators’: 50, ‘min_samples_split’: 5, ‘min_samples_leaf’: 2, ‘max_features’: ‘log2’, ‘max_depth’: 3, ‘learning_rate’: 0.05}.

**Figure 4:**
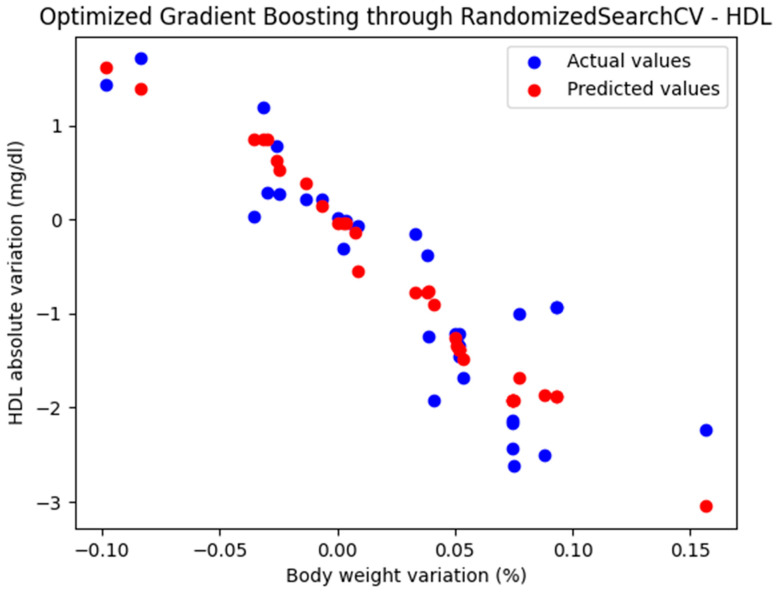
Comparison between the actual and predicted values obtained with the optimized Gradient Boosting model through RandomizedSearchCV.

For these hyperparameters, the graphical representation of the actual and predicted values obtained using the optimized Gradient Boosting model through RandomizedSearchCV is shown in Figure 4.

The optimized Gradient Boosting model, through RandomizedSearchCV, yielded the following performance metrics: MAE = 0.379, MSE = 0.235, and R^2^ = 0.823. With an R^2^ score of 0.823, the model demonstrated a very good fit and strong predictive performance.

Table 2 presents the performance of the models using GradientBoostingRegressor. It can be observed that the models optimized with GridSearchCV and RandomizedSearchCV exhibited similar performances, both of which are considered very good.

### 3.3. XGBoost

In the initial approach, the XGBRegressor function was used with the default parameters: n_estimators = 100, learning_rate = 0.1, and max_depth = 3.

**Figure 5:**
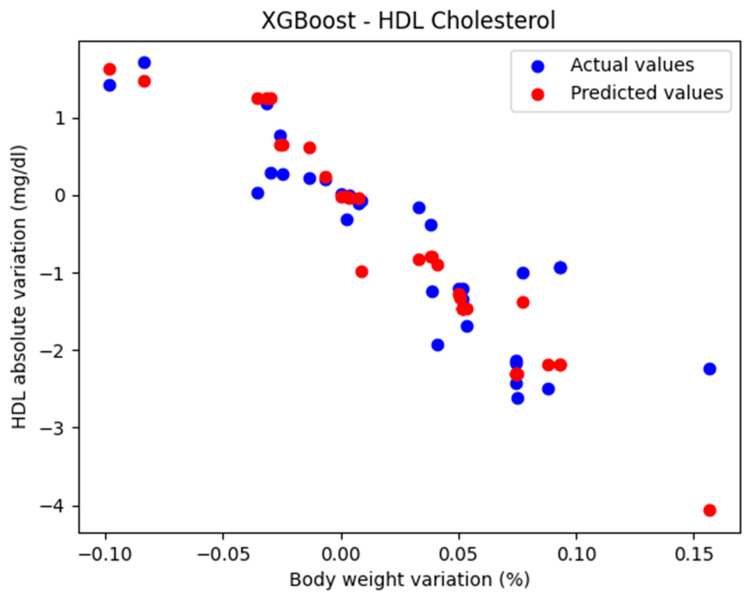
Comparison between the actual and predicted values obtained with the XGBoost model.

The comparison between the actual and predicted values using the XGBoost model, for the analysis of the absolute variation in HDL cholesterol relative to the percentage variation in body weight, is illustrated in Figure 5. The model evaluation yielded the following results: MAE = 0.424, MSE = 0.385, and R^2^ = 0.710. These findings support the classification of the model as good.

#### 3.3.1. Optimized XGBoost Model Through RandomizedSearchCV

Due to the small sample size, the implementation of XGBoost requires a conservative hyperparameter configuration aimed at mitigating overfitting and promoting model generalizability to broader, unseen datasets.

RandomizedSearchCV is a hyperparameter optimization technique that searches for the best combinations randomly, in a predefined hyperparameter space. The hyperparameters used in parameter grid had the following values: {‘n_estimators’: [50, 100], ‘learning_rate’: [0.1, 0.2], ‘max_depth’: [2, 3, 4], ‘min_child_weight’: [3, 5], ‘subsample’: [0.8, 1.0], ‘colsample_bytree’: [0.8, 1.0], ‘gamma’: [0.1, 0.5], ‘reg_alpha’: [0.1, 0.5],’reg_lambda’: [1.0, 2.0]}.

The best parameters obtained for XGBoost model through RandomizedSearchCV had the following values: {‘subsample’: 0.8, ‘reg_lambda’: 1.0, ‘reg_alpha’: 0.1, ‘n_estimators’: 50, ‘min_child_weight’: 3, ‘max_depth’: 3, ‘learning_rate’: 0.2, ‘gamma’: 0.1, ‘colsample_bytree’: 0.8}.

The optimized XGBoost model, through RandomizedSearchCV, yielded the following performance metrics: MAE = 0.402, MSE = 0.302, and R^2^ = 0.772. With an R^2^ score of 0.772, the model demonstrated a very good fit and strong predictive performance.

**Figure 6:**
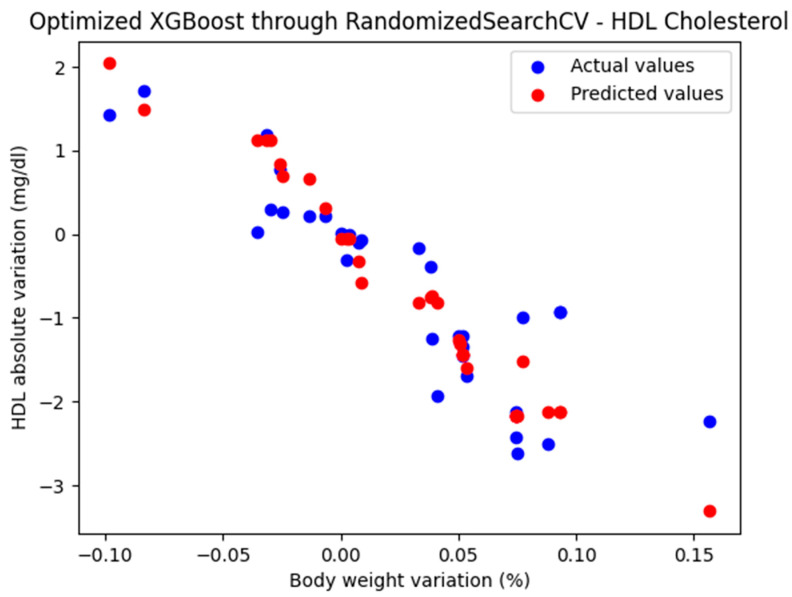
Comparison between the actual and predicted values obtained with the optimized XGBoost model through RandomizedSearchCV.

The representation of the actual and predicted values obtained from the optimized XGBoost model through RandomizedSearchCV, which was used to analyze the absolute variation in HDL cholesterol relative to the percentage change in weight, is shown in Figure 6.

#### 3.3.2. Optimized XGBoost Model Through GridSearchCV

Given the small size of the dataset, hyperparameter tuning was performed using GridSearchCV with appropriate values for the parameter grid: {‘n_estimators’: [50, 100, 150], ‘max_depth’: [3, 5], ‘learning_rate’: [0.05, 0.1, 0.2], ‘subsample’: [0.8, 1.0], ‘colsample_bytree’: [0.8, 1.0]}.

The best hyperparameters obtained for the XGBoost model through GridSearchCV were {‘colsample_bytree’: 0.8, ‘learning_rate’: 0.05, ‘max_depth’: 3, ‘n_estimators’: 50, ‘subsample’: 0.8}

**Figure 7:**
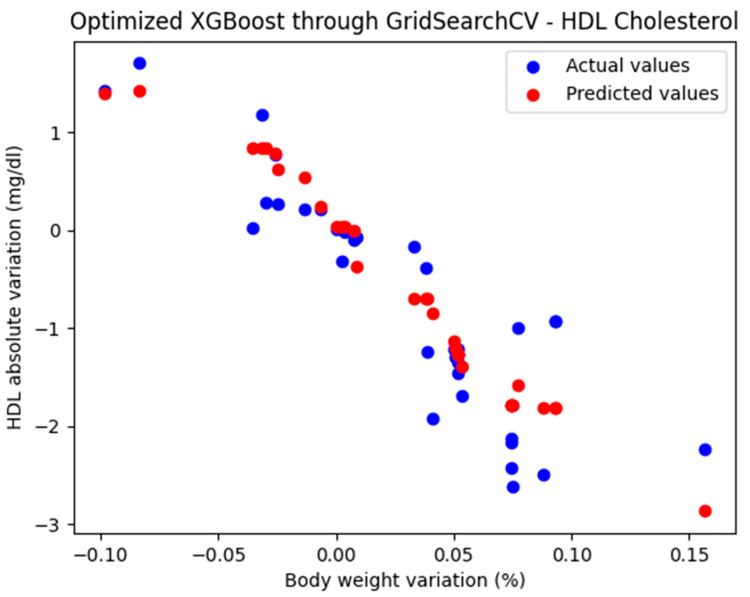
Comparison between the actual and predicted values obtained with the optimized XGBoost model through GridSearchCV.

For these hyperparameters, the graphical representation of the actual and predicted values obtained using the optimized XGBoost model through GridSearchCV is shown in Figure 7.

The optimized XGBoost model, through GridSearchCV, yielded the following performance metrics: MAE = 0.385, MSE = 0.236, and R^2^ = 0.823. With an R^2^ score of 0.823, the model demonstrated a very good fit and strong predictive performance.

Table 3 presents the performance of the models using XGBRegressor. It can be observed that the models optimized with GridSearchCV and RandomizedSearchCV exhibited similar performances, both of which are considered very good.

The best performance, in terms of the R^2^ score, was achieved by the Gradient Boosting model optimized using GridSearchCV (score = 0.825), closely followed by the XGBoost model optimized with RandomizedSearchCV and the Gradient Boosting model optimized with RandomizedSearchCV (both with a score of 0.823). The models based on second-degree polynomial regression (score = 0.801), as well as those based on linear regression (score = 0.770), can be considered good to very good.

## 4. Discussion

Healthcare professionals play a crucial role in supporting people with obesity through stigma-free, biology-informed dialogue. Initiating supportive conversations, setting realistic weight loss goals, and providing proactive guidance can enhance motivation and self-efficacy. Given the biological challenges of weight loss, tailored support is essential [12]. Identifying motivated patients and providing tailored interventions can improve outcomes. Weight loss is more successful with a trusted clinician who offers supportive, non-judgmental guidance [8].

A 2008 study found that diabetic patients who experienced a 0.7% decrease in HbA1c while receiving oral hypoglycemic treatment had fewer cardiovascular events, a reduced need for retinal photocoagulation, and a lower likelihood of progressing to end-stage renal disease [13].

Predicting the percentage and absolute variation in various parameters depending on the percentage variation in weight has the role, for the patient, of motivating them to lose weight in order to improve their health status, and for doctors, it is a useful tool in making lifestyle and treatment recommendations for patients.

The variations in parameters that are improved by weight loss are summarized below:

(a) Blood glucose levels:
  - Fasting blood glucose: Weight loss can lower fasting blood glucose levels, reducing the risk of type 2 diabetes (if prediabetic) or complications of diabetes. For every 5–10% reduction in body weight, fasting blood glucose levels decrease by 5–20 mg/dL.
  - HbA1c: Tends to improve with weight loss. A 5–10% weight loss can reduce HbA1c by 0.5–2.0%.
(b) Lipid profile:
  - Total cholesterol: Weight loss generally reduces overall cholesterol levels. A 5–10% weight loss can lower total cholesterol levels by 5–12 mg/dL.
  - LDL cholesterol (‘bad cholesterol’): Decreases with weight loss, improving cardiovascular health. A 5–10% weight loss can lower LDL levels by 5–10 mg/dL.
  - HDL cholesterol (‘good cholesterol’): Typically increases with weight loss. HDL cholesterol levels increase by about 1–3 mg/dL for every 5–10% weight loss.
  - Triglycerides: triglyceride levels decrease significantly—by about 20 mg/dL or more—for every 5% reduction in body weight.
(c) Inflammatory markers:
  - C-reactive protein (CRP): Lower CRP levels reflect reduced systemic inflammation after weight loss. A 10% weight loss can result in a 25–30% reduction in CRP levels.
  - Interleukin-6 (IL-6) and tumor necrosis factor-alpha (TNF-α): weight loss of 10% or more can reduce these inflammatory markers by 10–20%, depending on baseline levels.
(d) Liver enzymes (can also be considered inflammatory markers):
  - ALT (Alanine Aminotransferase) and AST (Aspartate Aminotransferase): Weight loss improves these markers, indicating better liver health. A 5–10% weight loss can decrease ALT and AST levels by 20–30%.
(e) Hormones:
  - Leptin: Decreases with weight loss, reflecting a reduction in fat mass. Leptin levels decrease in proportion to the reduction in fat mass, often by 20–30% for a 10% weight loss.
  - Adiponectin: increases by 20–30% with a 10% weight loss, improving metabolic health.
  - Thyroid hormones (e.g., TSH): thyroid hormone levels may normalize with a 5–10% weight loss in individuals with obesity-induced thyroid dysfunction.
(f) Blood pressure and related markers: although they are not direct blood markers, systolic and diastolic blood pressure decrease by approximately 1 mmHg for each kilogram of weight loss (this finding was made by studying the variation in blood pressure depending on the absolute variation in weight.
(g) Markers of renal function: Creatinine and glomerular filtration rate. Improved weight management may improve renal function. It is more difficult to draw a conclusion in quantitative terms.
(h) Uric acid: a 10% weight loss can lower uric acid levels by approximately 0.5–1.3 mg/dL.

Based on the implemented models, it was found that greater improvements were observed in the case of moderate weight loss (5–10% of body weight).

A limitation of this study is the small number of records (between 107 and 138, depending on the analyzed parameter). Therefore, in the models based on Gradient Boosting and XGBoost, the hyperparameters were tuned to values suitable for the small dataset to prevent overfitting and ensure that the results could be generalized to larger populations.

Weight loss has beneficial effects: In diabetes, it helps prevent complications, while in prediabetes, it can prevent progression to diabetes [14]. Lifestyle modification remains difficult to implement and sustain for both patients and healthcare professionals, due to the multifactorial influence of behavioral, psychological, environmental, and social determinants on long-term adherence [15]. Modern lifestyle modification strategies for weight loss include mobile health applications [16], AI-driven chatbots [17], telemedicine [18], digital cognitive behavioral therapy [19], gamification techniques [20], and personalized interventions.

Regardless of the treatment strategy, a weight loss of approximately 10% provides the greatest preventive benefit against the development of diabetes in individuals with prediabetes or metabolic syndrome [21].

Based on the analysis of all prediction models, it can be concluded that weight loss can significantly improve several blood parameters, especially those related to metabolic and cardiovascular health.

The application proposed by our team provides additional motivation for weight loss by offering a quantitative representation of improvements in metabolic status following the effort involved in weight reduction.

## 5. Conclusions

In conclusion, the integration of model-derived estimates for individual blood parameters based on changes in body weight can serve as an effective tool for clinical communication. By presenting personalized and data-driven insights, physicians are better equipped to motivate patients to adopt healthier lifestyle habits that facilitate weight loss and, ultimately, lead to improvements in metabolic health.

## Figures and Tables

**Table 1:** The performances of models using linear regression and polynomial regression for predicting the values of absolute variation in HDL cholesterol depending on percentage variation in body weight.

Metric/Method	Linear Regression	2nd-Degree Polynomial Regression
MAE	0.447	0.441
MSE	0.434	0.263
R^2^ Score	0.770	0.801

**Table 2:** The performance of Gradient Boosting model with default parameters (GBR), optimized Gradient Boosting model through GridSearchCV (GBR_GS), and optimized Gradient Boosting model through RandomizedSearchCV (GBR_RS).

Metric/Method	GBR	GBR_GS	GBR_RS
MAE	0.437	0.374	0.379
MSE	0.424	0.233	0.235
R^2^ Score	0.694	0.825	0.823

**Table 3:** The performance of XGBoost model with default parameters (XGB), optimized XGBoost model through GridSearchCV (XGB_GS), and optimized XGBoost model through RandomizedSearchCV (XGB_RS).

Metric/Method	XGB	XGB_GS	XGB_RS
MAE	0.424	0.402	0.385
MSE	0.385	0.302	0.236
R^2^ Score	0.710	0.772	0.823

## Data Availability

Data are publicly unavailable due to privacy restrictions.

## Abbreviations

The following abbreviations are used in this manuscript:

MSE	Mean Squared Error
MAE	Mean Absolute Error
HbA1c	Glycosylated hemoglobin
HDL	High-Density Lipoprotein

## References

[1] DeFronzo R.A., Ferrannini E., Groop L., Henry R.R., Herman W.H., Holst J.J., Hu F.B., Kahn C.R., Raz I., Shulman G.I. (2015). Type 2 diabetes mellitus. Nat. Rev. Dis. Primers.

[2] Bays H.E. (2023). Why does type 2 diabetes mellitus impair weight reduction in patients with obesity? A review. Obes. Pillars..

[3] Stanford J., Kaiser M., Ablah E., Dong F., Paull-Forney B., Early J. (2012). The effect of weight loss on fasting blood sugars and hemoglobin A1c in overweight and obese diabetics and non-diabetics. J. Diabetes Mellit..

[4] Stratton I.M., Adler A.I., Neil H.A., Matthews D.R., Manley S.E., Cull C.A., Hadden D., Turner R.C., Holman R.R. (2000). Association of glycaemia with macrovascular and microvascular complications of type 2 diabetes (UKPDS 35): Prospective observational study. BMJ.

[5] Chandrasekaran P., Weiskirchen R. (2024). The Role of Obesity in Type 2 Diabetes Mellitus-An Overview. Int. J. Mol. Sci..

[6] Franz M.J. (2017). Weight Management: Obesity to Diabetes. Diabetes Spectr..

[7] Lopez-Jimenez F., Almahmeed W., Bays H., Cuevas A., Di Angelantonio E., le Roux C.W., Sattar N., Sun M.C., Wittert G., Pinto F.J. (2022). Obesity and cardiovascular disease: Mechanistic insights and management strategies. A joint position paper by the World Heart Federation and World Obesity Federation. Eur. J. Prev. Cardiol..

[8] Safaei M., Sundararajan E.A., Driss M., Boulila W., Shapi’I A. (2021). A systematic literature review on obesity: Understanding the causes & consequences of obesity and reviewing various machine learning approaches used to predict obesity. Comput. Biol. Med..

[9] Kirk D., Kok E., Tufano M., Tekinerdogan B., Feskens E.J.M., Camps G. (2022). Machine Learning in Nutrition Research. Adv. Nutr..

[10] American Diabetes Association Professional Practice Committee (2024). 8. Obesity and Weight Management for the Prevention and Treatment of Type 2 Diabetes: Standards of Care in Diabetes-2024. Diabetes Care.

[11] Churuangsuk C., Hall J., Reynolds A., Griffin S.J., Combet E., Lean M.E.J. (2022). Diets for weight management in adults with type 2 diabetes: An umbrella review of published meta-analyses and systematic review of trials of diets for diabetes remission. Diabetologia.

[12] Dicker D., Alfadda A.A., Coutinho W., Cuevas A., Halford J.C., Hughes C.A., Iwabu M., Kang J.-H., Nawar R., Reynoso R. (2021). Patient motivation to lose weight: Importance of healthcare professional support, goals and self-efficacy. Eur. J. Intern. Med..

[13] Gæde P., Lund-Andersen H., Parving H.-H., Pedersen O. (2008). Effect of a multifactorial intervention on mortality in type 2 diabetes. N. Engl. J. Med..

[14] Wilding J.P. (2014). The importance of weight management in type 2 diabetes mellitus. Int. J. Clin. Pract..

[15] Espinosa-Salas S., Gonzalez-Arias M. (2025). Behavior Modification for Lifestyle Improvement. StatPearls [Internet].

[16] Ghelani D.P., Moran L.J., Johnson C., Mousa A., Naderpoor N. (2020). Mobile apps for weight management: A review of the latest evidence to inform practice. Front. Endocrinol..

[17] Oh Y.J., Zhang J., Fang M.L., Fukuoka Y. (2021). A systematic review of artificial intelligence chatbots for promoting physical activity, healthy diet, and weight loss. Int. J. Behav. Nutr. Phys. Act..

[18] Ufholz K., Bhargava D. (2021). A Review of Telemedicine Interventions for Weight Loss. Curr. Cardiovasc. Risk Rep..

[19] Rychescki G.G., Dos Santos G.R., Bertin C.F., Pacheco C.N., Antunes L.d.C., Stanford F.C., Boaventura B. (2024). Online Cognitive-Behavioral Therapy-Based Nutritional Intervention via Instagram for Overweight and Obesity. Nutrients.

[20] Kurtzman G.W., Day S.C., Small D.S., Lynch M., Zhu J., Wang W., Rareshide C.A.L., Patel M.S. (2018). Social Incentives and Gamification to Promote Weight Loss: The LOSE IT Randomized, Controlled Trial. J. Gen. Intern. Med..

[21] Grams J., Garvey W.T. (2015). Weight Loss and the Prevention and Treatment of Type 2 Diabetes Using Lifestyle Therapy, Pharmacotherapy, and Bariatric Surgery: Mechanisms of Action. Curr. Obes. Rep..

